# Integrated health Services for Children: a qualitative study of family perspectives

**DOI:** 10.1186/s12913-021-06141-9

**Published:** 2021-02-23

**Authors:** Rose-Marie Satherley, Raghu Lingam, Judith Green, Ingrid Wolfe

**Affiliations:** 1 Department of Psychological Interventions, University of Surrey, Guildford, England; 2 Department of Women’s and Children’s Health, King’s College London, London, England; 3 Population Child Health Clinical Research Group, School of Women & Children’s Health, University of New South Wales, Sydney, Australia; 4 Wellcome Centre for Cultures & Environments of Health, University of Exeter, Exeter, England

**Keywords:** Child health, Integrated care, Health systems, Long-term condition

## Abstract

**Background:**

There is increasing evidence that integrated care improves child related quality of life and reduces health service use. However, there is limited evidence on family perspectives about the quality of integrated care for children’s services. This study aimed to understand children, young people, and caregivers’ perceptions of a new integrated care service, and to identify essential components of integrated care for children and young people with ongoing conditions.

**Methods:**

A qualitative analysis of in-depth interviews with caregivers and children included families (*N* = 37) with children with one of four ongoing conditions (asthma, eczema, epilepsy, constipation) who had experienced a new integrated care service delivered in South London, UK.

**Results:**

Four key components of integrated services identified were: that the key health-worker understood the health needs of the family in context; that professionals involved children and caregivers in treatment; that holistic care that supported the family unit was provided; and that families experienced coordination across health, social, and education systems.

**Conclusions:**

Children and families identify care navigation and a holistic approach as key components that make high quality integrated care services. Service developments strengthening these aspects will align well with family perspectives on what works and what matters.

**Supplementary Information:**

The online version contains supplementary material available at 10.1186/s12913-021-06141-9.

Chronic diseases are increasing in prevalence among children, often with poor outcomes as health systems struggle to meet children’s needs [1, 2]. Care is typically required from multiple health providers, sharing responsibility for a patient’s care across multiple settings, including schools. To address these issues, integration of health services is a policy priority [3]. The World Health Organization defines integrated care as: “*health services organized and managed so that people get the care they need, when they need it, in ways that are user-friendly* [4].” In the UK context, key issues of children’s services integration are vertical integration of accessible generalist primary care and paediatric expertise [5, 6] and horizontal integration of health and other services [3]. Integrated processes require coordinated care, generally involving a multi-disciplinary team, signposting, navigation, or pathways to ensure access to other services including non-urgent specialist care, enabled by organisational arrangements, financial levers, shared data and inter-operable technology such as electronic health records [7, 8].

Interventions to achieve these aims are diverse, with integration referring to a variety of service and system level interventions [9], with distinct types of integration relating to organisational features and the processes of delivering care [3, 10]. Given the range of interventions, often unclear definitions, complex design of integrated services, and variable understanding of what components constitute integrated care, evidence for the impact of integrated care is variable [11–14]. One systematic review, for instance, found evidence of increased service access, perceived care quality and patient satisfaction, but unclear evidence for other outcomes [12]. With respect to integrated care in children, a recent meta-analysis found that integrated care can be associated with greater improvements in children’s quality of life when compared to those receiving standard care, but that the lack of high-quality evidence from trials, inadequately theorised interventions and variable outcomes made it difficult to make recommendations about the effectiveness of integration [11]. However, there have been encouraging results on the impact of integration on reduction of health service use in children from service-based evaluation studies [5, 15, 16].

The policy drivers of integration may be around optimising service use and the efficiency of the health care system; however, a patient centred approach is central to achieving these aims [17]. One underlying assumption of integration is that it will result in patients experiencing “joined up” care, across professionals and providers, services and organisations. Given the importance of the patient experience as an outcome of integrated care for its own sake, as well as a potential mechanism to changes in service use or clinical outcomes, the perceptions of children and young people receiving integrated care, and their caregivers, are an essential indicator of care quality [18, 19].

This study aimed to identify the essential components of integrated care from the perspectives of children and families, in an ethnically and economically diverse setting in south London, UK. It drew on data from the implementation an integrated care programme: The Children & Young People’s Health Partnership (CYPHP)’s Evelina London model [20, 21]. Here, we do not aim to evaluate the service, but rather use family perspectives from an early trial phase to identify the key components of integrated care they attributed as essential for acceptability, appropriateness and effectiveness to inform future roll-out of this and other integrated care services for children.

## Methods

### Design

A qualitative interview study of participants who had experienced the early phase of a new integrated service was undertaken from June 2018–March 2019.

### Setting

The Evelina CYPHP approach is being trialled for children with ongoing conditions in south London, a highly diverse inner-city setting. The early phase of this integrated care project includes children with one or more of the following conditions: constipation, eczema, asthma, or epilepsy. The roll out of CYPHP was phased, allowing an opportunistic randomised control trial, and nested implementation and economic evaluation studies, to be conducted, prior to the approach being adopted as part of routine care.

CYPHP aims to strengthen the local health system through better integrated care. At the organisational level, a novel population health management (PHM) system is used to identify eligible children from primary care records, then invite children and families to complete a biopsychosocial pre-assessment, via a cloud-based portal. The information is used to triage and tailor early intervention care which includes health promotion and, when required, holistic care specifically for the needs of the child and family. Care is provided by a multidisciplinary health team consisting of specially trained Children’s Nurses, General Practitioners, Paediatricians, and mental health specialists. Most care is delivered by Children’s Nurses who provide a first point of contact for families, act as care navigators, coordinate and deliver care across primary, community, and hospital settings. The entire multidisciplinary team is available when needed, working closely with primary and community services, and standard referral pathways are used for specialist care. All clinical information regarding services delivered by CYPHP is held on shared electronic health records. Further details of the service can be found in recent publications [20, 21].

### Recruitment and sample

The PHM system enables all eligible children to be contacted proactively and, together with those actively referred to the service, these children and their parents or carers are invited to complete the biopsychosocial pre-assessment and participate in ongoing research. Preliminary analyses of those completing the preassessment (*n* = 219) indicate that a substantial proportion of children (60–76%) reported uncontrolled symptoms for asthma, constipation or eczema, and 28% reported high to very high scores on mental health symptom questionnaires [16]. Additionally, 68% of the cohort were from ethnic minorities. For this nested study, children and caregivers receiving the CYPHP service, who had identified that they were interested in participating in research, were eligible for recruitment.

A researcher contacted families, via phone, once they had received care for at least 2 weeks. The researcher was independent of the clinical service and had no previous contact with families. Families were fully informed about the aims of the study and were invited to take part in an interview on their experiences of the service. Out of the initial 40 parents contacted, thirty-seven caregivers gave written consent; children and young people over 12 years of age provided written assent. Recruitment continued until data saturation, which was established when additional new information from interviews was no longer obtained.

Caregivers could take part in interviews without their child present; all children took part with their caregiver present. Maximum variation sampling was used to ensure a range of child age, sex, condition, and socioeconomic status.

### Data collection

Demographic data relating to the child’s age, condition, time in service, and index of multiple deprivation (IMD) of home address was obtained. The IMD is the official measure of deprivation across England describing relative deprivation for small areas or neighbourhoods (containing on average 650 households) called lower-super-output areas [22]. IMD is calculated from seven distinct domains of deprivation, encompassing a wide range of living conditions; areas are ranked and assigned deciles ranging from the most deprived areas (1) to least deprived (10).

Interviews lasted between 40 and 60 min and were completed at a local clinic (*N* = 2) or in the family home (*N* = 35). Interview guides were developed with involvement from public and patient involvement groups and healthcare providers and adapted for different age groups; questions focussed on experiences of the integrated care service and symptom management. The main interview guide for this study is provided in Additional file 1.

A range of art-based methods were used to engage younger children in the discussions, asking children to construct a pictorial timeline or collage of how they felt before, during and after involvement in the integrated service [23]. Children spoke to the researchers as they created their work, and once finished, children were asked to talk about their artwork.

### Analysis

Interviews were audio recorded and transcribed verbatim. Analysis was directed at qualitative description, a method which stays close to participants’ own accounts [24], using thematic content analysis drawing on techniques from grounded theory traditions [25] with the aid of qualitative software (NVivo Version11). This entailed three researchers separately coding transcripts in an iterative process that combined deductive coding (from themes in integrated care literature) and inductive coding from comparisons within and across the data set. After coding a subset of the data, an initial set of codes was discussed by the research team, and compared within and across interviews, to identify patterns and amalgamate into themes. The research team met regularly to discuss and revise the emergent themes and ensure analytical rigour, with any discrepancies discussed to ensure analysis captured all perspectives.

### Ethical approval

Ethical approval was granted by the Health Research Authority (Reference: 17/SW/0275).

### Findings

Thirty-seven families completed interviews (19 patients, 40 caregivers and 8 siblings). Children and young people were between 29 weeks and 15 years of age (48.6% male). Sociodemographic characteristics of participants are detailed in Table 1: of the sample, 35.1% were from the 20% most deprived areas within the UK.

**Table 1:** Characteristics of Interview Participants

Child’s Age Band	Caregiver or Child Number	Index of Multiple Deprivation Decile (1-10^**a**^)	Family Present in Interview	Child Present in Interview	Child’s Condition	Duration of Contact with Service at Point of Interview
**Pre-School (0–4 years)**	1	3	Mother	No	Eczema	25 weeks
	2	5	Mother	No	Eczema	32 weeks
	3	3	Mother	No	Eczema	41 weeks
	4	5	Mother	No	Eczema	19 weeks
	5	3	Mother; Father	No	Epilepsy	7 weeks
	6	2	Mother	No	Epilepsy	29 weeks
	7	2	Mother	No	Constipation	17 weeks
	8	7	Mother	No	Epilepsy	3 weeks
	9	2	Mother	No	Eczema	7 weeks
	10	5	Mother	Yes	Constipation	3 weeks
	11	3	Mother	No	Epilepsy	4 weeks
	12	4	Mother	Yes	Constipation	5 weeks
	13	7	Mother	Yes	Constipation	3 weeks
	14	2	Mother	Yes	Constipation	11 weeks
**Primary School (5–10 years)**	15	2	Mother	No	Asthma	71 weeks
	16	1	Mother; 1 sibling	Yes	Eczema	12 weeks
	17	4	Mother; 1 sibling	Yes	Constipation	29 weeks
	18	2	Mother	No	Constipation	6 weeks
	19	4	Mother	Yes	Asthma	24 weeks
	20	6	Mother	Yes	Epilepsy	8 weeks
	21	4	Mother	No	Epilepsy	3 weeks
	22	2	Mother; 1 sibling	Yes	Eczema	27 weeks
	23	2	Mother; 2 siblings	No	Eczema	24 weeks
	24	1	Mother	Yes	Eczema and asthma	19 weeks
	25	2	Mother	Yes	Epilepsy	3 weeks
	26	4	Mother	No	Asthma	8 weeks
	27	5	Mother; 1 sibling	Yes	Asthma	10 weeks
**Secondary School (11–16 years)**	28	2	Mother	No	Asthma	11 weeks
	29	1	Mother	Yes	Constipation	25 weeks
	30	8	Mother; Father	No	Constipation	42 weeks
	31	2	Mother	Yes	Asthma	31 weeks
	32	3	Mother	Yes	Constipation	35 weeks
	33	6	Mother; Father; 1 sibling	Yes	Epilepsy	44 weeks
	34	8	Mother	Yes	Asthma	32 weeks
	35	5	Mother; 1 sibling	Yes	Asthma	12 weeks
	36	2	Mother	No	Asthma	15 weeks
	37	6	Mother	Yes	Epilepsy	45 weeks

^**a**^ Index of Multiple Deprivation Decile ranges from 1 to 10. Deciles are calculated by ranking areas across England from most deprived (1) to least deprived (10) based on 2019 data

Exposure to the new service varied widely, depending on condition and needs (ranging between 3 and 71 weeks). Despite this, families described important commonalities of integrated care in combination with aspects of perceived quality and effectiveness of the service. Most families valued the integrated service they received, commenting on advantages over prior experiences of traditional services, their willingness to recommend the service to others, and support of continued funding for the service.

*“To the people in charge; you need to expand this service to everybody, everybody who has children with medical conditions, who need the help, who require the help.”* [Caregiver 14; Constipation]

Below, we describe these as four key themes (Table 2) defined by summary phrases derived from participants’ accounts. The first theme, The Nurse Understands Our World, comprised of four subthemes, demonstrates the importance of addressing broader determinants of health. The second theme was entitled, Professionals Involve Us in Treatment, and again was formed of four subthemes. This theme highlights the importance of relationships with key providers, developed through physical presence and supplemented by technology. The third theme, Professionals Support All Our Needs, comprised of three subthemes, speaks to the importance of holistic care provided by a key professional but supported by the wider health system. The final theme entitled The System is Glued Together, formed of five subthemes, speaks to the value of a health system that is perceived as coordinated, across health, social and education sectors. The following sections describe these themes and associated subthemes in detail. Themes and Subthemes are presented in Table 2; the thematic analysis developed integrates subthemes to create a coherent narrative.

**Table 2:** Overview of themes and sub themes with exemplar quotes

Theme	Subtheme	Illustrative quotes
The Nurse Understands Our World	Initial Distrust of Intrusion	*“Well at first, I was a bit unsure, just wondering whether she was here to judge, or you know, even tell you off. It was just a bit unusual to have someone come in and ask all about your actual home and life.”* (Caregiver 16; Constipation) *“[Nurse] was very full-on and very in your face after the first meeting … very personal stuff, it was like, whoa, slow down! You’re here for her asthma and we need to trust you before anything more, it’s all a bit quick and intrusive.”* (Caregiver 19; Asthma) *“It felt a bit, I wouldn’t say uncomfortable but more interesting … why am I talking to [Nurse] … about my daily life or something, instead of talking about my asthma.”* (Child 34; Asthma)
	Time Facilitates Personal Disclosures	“*It always felt like a conversation … [Nurse] made it so relaxed, she took time to hear what we was going through and that let us open up more than I have with any other medical person*.” (Caregiver 3; Eczema) “*It just felt much calmer and natural and easy, and [Nurse] was so lovely so friendly. She had all the time that we needed, she was in no hurry at all, she was really interested in us. She got to know us personally, bit by bit.”* (Caregiver 16; Constipation) “*In hospitals you can’t really trust anyone because they’re all strangers, but I knew when I first come here that everyone here’s a stranger to me but once I had like one or two sessions with everyone here it’s like I felt like I can trust them all.”* (Child 32; Constipation)
	Feeling Heard and Receiving Contextualized Support	*“[Nurse’s] are the only two that have not lectured me about my Dad smoking. So when you go to the hospital they tend to sort of look at you and kind of go, oh well you know, you need to, you need to nag your dad and I’m like but it’s his house, I can’t, I’m living in his space, unfortunately I can’t change that. But [Nurse’s] listen … they try and work around it, or work with it.”* (Caregiver 19; Asthma) “*It was nice of [Nurse] to come here and talk to me and [Child], especially [Child] and to actually see him understand, hear him out.”* (Caregiver 22; Eczema)
	Material Resources that Flow (or not) from Being Heard	*“When those vouchers come every month it helps, without those we can’t eat the veg … I don’t think I could have got that on my own … the [service] is fantastic in that sense”* (Caregiver 7; Constipation) “*I’m suffering with mold, can you help me out, and she said, yeah, just go to this website, and you can call them... I did end up calling them*.” (Caregiver 9; Eczema) *“[Nurse] did get us the bed alarm, so it alerts us if there’s a bedtime seizure.”* (Caregiver 37; Epilepsy)
Professionals Involve Us in Treatment	Importance of Relationships with Key Professional who Coordinates Care	“*This scheme has been much more two-way, it’s been amazing, you know, I’ve always been able to contact [Nurse], she has checked in with me to see how things are, and that makes such a big difference*.” (Caregiver 33; Eczema) *“[Nurse] is always there, on the phone or the email, it’s more like a grandma. Someone to say “Oh I’m worrying about this”, and she sets us on the right path, she knows what we need.”* (Caregiver 35; Asthma)
	Respect for Young Person’s Autonomy	“*The way they do it is to get her to understand, they do it in the kiddie way, which is great because now she’s more interested, she wants to listen, she wants to know what’s going on.*” (Caregiver 19; Asthma) “*I know the plans, because mummy and [Nurse] taught me them, and I can just do it now. I might not like doing it all the time, but I can, and it makes me feel normal, and I’m like the one, you know, doing it*.” (Child 24; Eczema and Asthma)
	Trust is Built on the Recognition of Professional Learning and Enhanced Through Physical Presence	*“[The Nurse] is continually learning … she was telling me about talks she’d been to, she’d ring me and say ‘you know, this book might be interesting for [Child]’. And that kind of reassured me that she knew her stuff.” (Caregiver 16; Constipation)* “*Because [Nurse] knows us well, he quickly texted me back telling what I needed … it’s a quick text message now because he’s seen our story.”* (Caregiver 16; Asthma) *“[Nurse] knows me now and I’m not got so many questions. So we can just text now if I need to ask her about something.”* (Child 33; Epilepsy) “*It’s much more organic, I feel much more relaxed, you’re more prepared to question things rather than just accept … I think, because someone has put themselves physically out there for you it’s noticeable*.” (Caregiver 33; Epilepsy)
	Negotiating Point of Independence	*“We don’t want to lose the service but obviously you can’t have everyone staying on it forever, I don’t know if it would be possible just to sort of ease it down over the time, so it was more like, it’s still there if you need it.”* (Caregiver 2; Eczema) “*She’s obviously given me the choice whether I want to keep her on or not, but as I said she’s been fine, I know what I’m doing with her*.” (Caregiver 10; Constipation) *“I said, “Don’t leave us”, I said, because the four seasons, that’s when her asthma plays up and once we’re on our own we’re off to the doctor or off to the A&E, so I said, “Don’t …*”*, you know, “Stay, because then you can help.”* (Caregiver 36; Asthma)
Professionals Support All Our Needs	Looking After the Whole Person	*“[the Nurse is] even asking like about behaviour, how is his behaviour, how is his sleep pattern, so it was kind of that general getting a picture of [Child].”* (Caregiver 17; Constipation) “*It’s been really amazing, from where I was when my anxiety was bad, my asthma was bad, and then having them to help me through it was really, really helpful*.” (Child 34; Asthma)
	Importance of a Multidisciplinary Team Beyond the Key Professional	*“[Nurse] goes well I’ll try and get him assessed by [Mental Health] and I’m thinking to myself, don’t waste your time love … it came so quick and easy … I’m thinking wow these people really, they have what they need in place to help.”* (Caregiver 15; Asthma) *“[Child] explained to [Nurse] how, what he was going through, you know, something he took on board and then [Nurse] went back and spoke to the pharmacist and discussed other alternatives.”* (Caregiver 27; Asthma) *“And what helped her most is that [Nurse] work alongside [Mental Health Team], so she was aware of the problems that [Child] was having, which was quite helpful.”* (Caregiver 32; Constipation)
	Preference for Conventional Care^a^	“*I think the service is great as a whole, the only thing I would say would be the whole mental health side of things. You know, I think if someone says no, we’re great but thank you for your support, I think that should be enough and I think the focus should be on the child at hand*.” (Caregiver 19; Asthma)
A System that is Glued Together	Connecting Services Together	*“[Nurse] tried to help us navigate the systems to find something, so she got in touch with our social worker and said, ‘look, these people have been abandoned and they need help, we need to try and get them help’ so she helped us and sort of nagged our social worker to access the Adoption Support Fund”* (Caregiver 30; Constipation) “*Another real great use has been like linking up with other services, so we’ve been linking up with the allergy people at St Thomas’ and also the dermatology department and the GP and just linking it all together*.” (Caregiver 2; Eczema) “*It’s like in my social work days, it’s like joined up thinking between different agencies was always important and I think now, to join up that conversation with the school and other people, you know, you see the sense in it.”* (Caregiver 33; Epilepsy)
	Importance of Formal and Informal Contacts	“*[GP] must see the records from the [Nurse] because then I ring saying, “Oh [Nurse has] recommended prescribing Eumovate” and they say, “Oh yeah, we see that” and then they’ll just do it*.” (Caregiver 2; Eczema) *“[Nurse said] don’t worry, leave it with me, I will deal directly with them and I will put it on to the system, and then it was on the system, he got the prescription on the system. Easy.”* (Caregiver 15; Asthma)
	Communication in the Context of Uncertainty^a^	*“The communication between you guys … and the GPs probably need to be strengthened, but it’s more from the GP’s side if that makes sense. [the Nurse] would suggest all these things but the GP says no. So we’re stuck again.”* (Caregiver 3; Eczema)
	Supporting All Sectors to Take on a Meaningful Role in Child Health	“A *few times she’s wanted to go [to the toilet] and the teachers have told her no. Where now, where [Nurse] got in contact, a medical professional, they’re listening so if she needs to go, she’s allowed to go now.”* (Caregiver 32; Constipation) *“[Nurse] was busy chasing up [CYP]’s school to speak to the school the nurse … so, school nurse went to [CYP]’s school and cleared out the cupboards, because certain things just wasn’t supposed to be there*.” (Caregiver 36; Asthma) *“I had to talk to the police for some stuff and she helped me work through that. And I think she may have told them about my epilepsy because they seemed to know. Yeh, at first, they thought I was faking when I was ill!”* (Child 37; Epilepsy)
	Challenges of Working Alongside a Stretched Workforce	*“[Nurse] was getting frustrated I believe [with GP]. I don’t want to speak for her because a lot of the things that she would suggest, she was like, “Has that been done yet?” “No.” “Okay, I’ll have to send another,” “Has that been done yet?” “No.”* (Caregiver 3; Eczema) *“[Nurse] and [Mental Health Nurse] both of them have constantly called the school, they’ve tried liaise, sometimes they can’t get through, sometimes they leave messages, the teachers don’t get back*.” (Caregiver 32; Constipation) “We normally see [Nurse] in our GP but they, like she messages them asking for a room and they don’t reply, so that’s why we can’t really see her as much as we often did.” (Child 32; Constipation)

^a^These subthemes represent discrepant or few cases that aided in the interpretation of analysis

### The nurse understands our world

A key component of integrated care that families reported as important was care that addresses their personal and social needs. Families in this study reported a variety of social needs including damp housing that exacerbated asthma, difficulties attending school due to ongoing symptoms, and challenges maintaining social relationships when supporting a child with an ongoing condition. Despite families emphasising the importance of discussing these needs, several acknowledged an initial reticence, describing questions around personal and social needs as “*intrusive*”. For example, one mother living in a socioeconomically deprived neighbourhood, reported fear of being “*penalized*” by the Nurse when asked about her home environment, considering these questions to be unrelated to her child’s health (Caregiver 23).

*“At first, I was a bit like is she [Nurse] was going to penalize us?... She’s coming in here to fix the eczema, why should she want to know this?”* (Caregiver 23; Eczema)

The consensus among families interviewed was that time with the Nurse was needed to facilitate the disclosure of personal information. Most families reported that they developed a level of “*trust*” with their Nurse. For example, one mother describes how, despite her initial concern around disclosing personal information, she felt able to disclose her needs once she had developed a relationship with her Nurse (Caregiver 30). However, in direct contrast, a second mother raised ongoing concerns about disclosing personal information (Caregiver 25). Caregiver 25 was the only participant to raise ongoing concerns around disclosing personal circumstances to the Nurse. These concerns surrounding the discussion of personal and social circumstances in a consultation about a health condition may reflect the traditional separation of health and social services.

“*It was weird at first, but I made us a cuppa tea, had a general chit-chat. And by the end of my first cup, I felt I could open up to her. Like I knew her more as a person*.” (Caregiver 30; Constipation)

“O*ur problems [is] her seizures, fix that … if she don’t want to speak about school, fine … if I don’t want to talk about my job, then leave it. What you do with that stuff anyway … I don’t want it on her record.*” (Caregiver 25; Epilepsy)

Most participants expressed views that discussion around the broader determinants of health, and time spent with providers, enabled them to feel understood by the Nurse. This understanding then facilitated the delivery of interventions suited to the family’s needs and contextualized to their social circumstances. A typical example is provided below, whereby the child’s Nurse supported him in his desire to play football, as an important health goal or outcome the child wanted to achieve (Child 27). Evidence that this support was a key component of integrated care comes from families’ descriptions of prior experiences with health providers whose advice felt inappropriately narrow and had not considered their wider needs.

*“One time they said it was running in my football and I should stop. But football is everything, I want to be a professional footballer and you’ve basically said I can’t do that [referring to previous experiences of health care]. But [Nurse] really understood and took that on board and said like how we can work with asthma and football.”* (Child 27; Asthma)

For some families, material benefits resulted from these discussions, including housing support, food vouchers, services, and goods. However, on occasion, expectations were raised about needs that could not be met. An example of this is provided by Caregiver 31, who described their Nurse’s challenges in obtaining the resources required to support the family in the context of a stretched housing sector.

*“So [Nurse] tried to push Council property, but they won’t give [a property to] me because they said oh too many people are in the queue, they won’t give me. [Nurse] tried hard and pushed, he tried.”* (Caregiver 31; Asthma)

Whether discussion of needs resulted in changes in social circumstances or not, the key was that families felt understood by their Nurse, and that this social contextualising of their child’s health was welcomed and valued by most families.

### Professionals involve us in treatment

Connected to the capacity of the Nurse to understand families’ worlds, the second theme relates to the nature of relationships that this connection engenders within the health care system. Most families, including those who were initially reticent, described a mutual trust that developed between themselves and the Nurse over time. This reciprocal trust appeared key to family engagement with the service. This manifested in participants feeling confident in asking questions about care, feeling able to suggest alternatives as well as receiving advice, and a sense of both parties (families and professionals) learning together. Numerous families reported a two-way process with the Nurse that enabled different understandings of conditions to be discussed, including beliefs around the urgency of treatment and appropriateness of medication. There was also a sense that the Nurse’s availability to families facilitated this collaborative approach (Caregiver 4).

*“If something happened, I could text her or call her and say, “this has happened, do you think I should do x, y or z?” We did that last week, texted her and we spoke together and came with a plan together.”* (Caregiver 4; Eczema)

Reciprocal relationships with professionals were also highlighted by older children as an essential component of their care. That this was attributed to integrated care was evidenced in their comparisons with previous services, in which children recalled feeling less involved in decision making. Older children reported that being involved in treatment allowed them to feel more agency and engagement with their care, highlighting the importance of respecting young people’s autonomy in treatment decisions (Child 34).

*“[Nurse and Mental Health Provider] actually talk to me before they talked to my parents, which I really liked because it meant that they got my permission first … I felt I was actually in control. I want to do this.”* (Child 34; Asthma)

These relationships with professionals and trust in the Nurse appeared to be built through experiences of professionals being transparent about mutual learning. Caregiver 32 provides a typical example of this.

*“[The Nurse] is continually learning, she was telling me about conferences she’d been to … and that kind of reassured me and let me trust her.”* (Caregiver 32; Constipation)

Physical presence, at least early in the relationship, was important. All families spoke of the importance of face-to-face consultations in facilitating the development of a relationship with their Nurse (Caregiver 22). Families reported that communications technologies (email, SMS, phone calls) maintained this relationship over time, but, importantly, these were only positively rated once carers had established a physical face-to-face relationship. The uncertainties recounted by one father (Caregiver 5), whose partner had more contact with health providers, gives an example of the importance of relationship-building.

“[*The Nurse] got to know us, like as people. She could see us and understand us. But she knows us now, knows what we need … so now we do it on the phone more.*” (Caregiver 22; Eczema)

“*I don’t have the rapport that [Partner] has because she’s the one that sees [The Nurse], so I don’t know how good [The Nurse] is in terms of that, you know, in terms of her overall knowledge and also what she’s allowed to advise on.*” (Caregiver 5; Epilepsy)

Most families described increasing independence in managing children’s health over time, where they could rely on ‘*remote*’, rather than in-person, consultancy from their Nurse:

*“I would only ever go to A&E now if either I couldn’t get hold of [The Nurse], or it was a horrendous one, other than that we’re kind of happy to have sort of remote consultancy.”* (Caregiver 5; Epilepsy)

However, as discharge from the service approached, families’ expectations varied, with half expressing readiness for independent self-management and others reticent about discharge from the service. As one child says, “*I know what I’m doing now, I’ve got control”* (Child 35; Asthma). By contrast, Caregiver 29 questioned the fairness of discharge from the service; despite her child’s needs being met, this mother wished to stay in contact with the service. This desire appeared prominent among families who were referred to the service with high levels of need as indicated by frequent use of emergency department services and concurrent mental health needs. For some families, the prospective withdrawal of the Nurse was framed as a withdrawal of care, rather than a marker of growing independence.

*“We’re doing everything anyway, we haven’t been to hospital … I just felt like … just stay around for a little bit just in case … to have all this and then get it taken away. That’s just not right.”* (Caregiver 29; Constipation)

Relationships with Nurses were facilitated through open communication, availability to families, shared learning, and face to face relationships. Ensuring active family involvement in decision making processes was key to success, supporting engagement with the service, independent self-management, and readiness for discharge.

### Professionals support all our needs

One of the key principles of integrated care is considering physical, social, and psychological needs. Most families described the integrated service as providing holistic care, within a family, rather than just focusing on a condition. One mother used the metaphor of the service creating a “*whole circle*” supporting her family.

*“It’s a support system, it is not only about the child or about the person who is having the issue, it’s about the whole circle that is surrounding them, so, which is not going to improve the life of only the person who is sick, but only, even the people that are surrounded in that circle.”* (Caregiver 18; Constipation)

Of note, this mother speaks of a “*system*”, highlighting the importance of a wider network of professionals who support the family. References to this “*system*” are voiced by another mother who describes her Nurse talking with a paediatrician about her child’s care, highlighting again the role of the multidisciplinary team in facilitating holistic care (Caregiver 2). Caregiver 32 highlights the perceived crucial role of the Nurse as the empathetic interface for the multidisciplinary team. Both listening to families and identifying and advising on a broad range of health-related issues, including health promotion, disease and complication prevention, and the Nurse provided early intervention, coordinated care and onward referral within the multidisciplinary team when required. Other families detailed the ways Nurses discussed and identified mental health needs, and successfully brought in the wider team for onward referral to specialist services.

*“Sometimes [Nurse] said she’s wasn’t sure. And so it’s good to know that there’s people she can talk to, things like when she needs a paediatrician, there’s even that.”* (Caregiver 2; Eczema)

*“[Nurse] was helping with all the anxieties, doing exercise, eating well, the anger but it got quite bad … She spoke to her team and that’s how [Mental Health Nurse] became involved … And what helped her most is that she [Mental Health Nurse] works alongside [Nurse], so she was aware of [CYP]’s problems.”* (Caregiver 32; Constipation)

Most interviewees were positive about these holistic aspects of integrated care and welcomed support for the whole family. However, for two families, this aspect of integrated care was perceived as unhelpful, and even unnecessary or time wasting. Both these families described considerable use of health services prior to referral to the integrated service, and their views are resonant with a biomedical focus, in which physical and mental health are separate domains:

“*You’ve got this mental health thing, oh my God. Great but not for us* … *I just wanted to get onto the point with asthma and that’s it*.” (Caregiver 26; Asthma)

Holistic care is, then, a core component of integrated care from families’ perspectives: rejected by a few who preferred a more biomedical approach but emphasised and valued by most families who welcome additional support beyond the presenting condition, reassurance for caregivers, and improving the skills of the whole family in condition management.

### A system that is glued together

The fourth theme is that of encountering a system which did not just address holistic needs, but also responded in a co-ordinated and coherent way. This is summarised as ‘a system that is glued together’, which reflects the ability of the integrated service to coordinate across primary and secondary care, as well as health, social, and educational sectors. Interviewees frequently described their Nurse’s ability to coordinate across providers, pulling information together, and enabling all professionals in the patient’s life to provide seamless support.

*“I think the service helped us socially by being present with the school and being a common point of contact with school, GP and hospital.”* (Caregiver 6; Epilepsy)

Most families described good communication between their Nurse, primary and secondary care, and other non-health sector services, which enabled coherent and coordinated care. Again, evidence that this was a feature of the integrated service was typically provided by comparisons of previous encounters with health and other providers (Caregiver 2). Families typically described the Nurse as ‘*talking*’ to other professionals to achieve this, however some families reported not quite knowing how communication was achieved. Several mothers described how this communication enabled efficient access to secondary care services, highlighting the Nurse’s role as patient advocate (Caregiver 4).

*“I don’t know how [Nurse] can manage to see what the GP is writing down because I’ve had so many problems where one doctor can see [Child’s] notes but the other can’t.”* (Caregiver 2; Eczema)

“[*Nurse] actually trying to get us seen by dermatology again this week … so she said she’s just talk with them [dermatology] and that’s it, done.”* (Caregiver 4; Eczema)

As a core component of integrated care, coordination was on occasion flagged because of its absence. Two examples of missing coordination were in the context of their children undergoing further diagnostic tests with multiple providers. This highlights the importance of Nurses with expertise in sharing information across providers, particularly during times of clinical uncertainty, which may require input from a range of providers across primary and secondary care. In addition, this demonstrates the value of coordination across sectors and providers for effective integrated care.

*“I was a bit baffled by her last consultant’s and GP appointment, they was saying different thing to me and I was saying to [the Nurse], it doesn’t make sense, that is confusing, it doesn’t go with what you said.”* (Caregiver 19; Asthma)

However, more commonly, coordination was recognised as more present in the integrated system than it had been in previous health services. Regarding the new service, families reported enhanced coordination across health providers, and between health and social sectors. One frequently reported example of the difference between an integrated service and a conventional service, was coordination between the health and education sectors, which enabled patients to follow treatment plans outside the home or hospital setting. This was facilitated by the Nurse supporting schools to take on a meaningful role in the child’s health, which appeared to be achieved through the provision of school and student training, enabling coordination across sectors (Child 33).

*“My teachers and friends all know what to do if something happens. So, I don’t worry about it. My mum and dad don’t worry about it. Because everyone knows what to do if something did happen. And that’s probably because they’ve all been taught about epilepsy and about my epilepsy.”* (Child 33; Epilepsy)

Despite families valuing coordination across sectors, some caregivers highlighted the challenges they, and their Nurse, had in coordinating care with both the education sector and across the primary-secondary interface. These difficulties highlight the challenges in working within the context of school and healthcare workforce constraints (Caregiver 19).

“[*Nurse*] *was waiting for school to get back about [Child] … I went in and said, oh by the way, the [Nurse] has been trying to contact to try and speak to you about [Child] and has had no response … they say they’re too busy.”* (Caregiver 19; Asthma)

Whether families experienced a coordinated service or experienced challenges with coordination across the system largely depended on how well health, social and education sectors were updated with children’s health records: a key aspect of integrated care.

## Discussion

Despite the wealth of calls for integration of complex systems to address the gaps in healthcare delivery for child health, there has been little evidence on what integrated approaches look like for children and young people [11]. This is the first study using robust qualitative research to document family perspectives on a new integrated service and to identify essential components of integrated care for children and young people. The four components identified as contributing to the quality of integrated care are: 1) that the nurse understood their world 2) that professionals involved the family in treatment; 3) that professionals provided holistic support to the family; and 4) that the system was ‘glued together’, in that professionals coordinated the existing health, social, and education systems.

A growing body of work speaks to the importance of positive social aspects of system integration [17]. The nurses’ role was key to the implementation of the CYPHP model. Within this it was trust between the family and the practitioner that seemed to be central. Trust is important in both effective relationships between patient and provider, and across the health system [26]. In their realist review, Tyler et al. highlight the importance of “bridgers” or “connectors” in enabling trust between families and the health system to foster integrated care [27]. Similarly, families in this study highly valued the relational aspects of integrated care; relationships with providers, and trust, were central to families’ descriptions of good care. Like Tyler’s findings, trust was embodied to some extent within the Nurse and the role he/she played in engaging the child and wider family in treatment, providing holistic care, and facilitating access to specialist care. Clinical processes that facilitated these relational aspects of care included increased time with, and ease of access to, the Nurse. Face-to-face consultations with the Nurse were important; once established, relationships could then be supplemented using communications technology. These findings support Vassilev’s argument that technology is one component of an integrated system that only delivers benefits to patients when it supports the development of established patient-provider relationships [28].

This work expands upon the work of Plochg and colleagues, who question whether integration can improve the performance of healthcare without breaking down current silos within the health system [29]. In the model studied here, such structural integration across primary and secondary care seemed manifest in families’ accounts of the value of their Nurse’s actions in care coordination. Families spoke of the Nurse advocating for their needs, working as part of a multidisciplinary team, across primary-secondary healthcare boundaries and across interfaces of health, social and educational services. Clinical processes enacted by the Nurse went beyond navigating or signposting to services or resources, for example Nurses advocated and through closer inter-sectoral collaboration enabled referral to additional services. Further work is needed to understand the complexities of implementing this way of working in which building family relationships and coordination with the wider health system seem crucial, to negate unintended consequences, for example on discharge from care.

Finally, this work highlights the contextual and organisational processes that contribute to perceptions of high-quality integrated care [9]. Families highlighted the importance of delivering care which takes their holistic needs and social contexts into account, thereby also addressing the wider determinants of their health. However, the identification of additional needs, in a financially stretched health, educational, and social care environment meant that identified needs outside the health service could not always be met. Care navigation may be a necessary condition for quality care from family perspectives, but it is not a sufficient condition. Challenges reflect difficulties in integrating across sectors, whilst operating in constrained economic conditions. Integrated care cannot, therefore, necessarily mitigate shortcomings elsewhere in-service provision and policy; broader system integration and whole society change is essential to improve the quality of life for children with long term conditions. For child health, inter-sectoral collaboration across health and education sectors was critical and facilitated through the family’s Nurse. These findings resonate with the wider literature, highlighting the importance of inter-sectoral collaboration as a key mechanism in ensuring the success of integrated child health programmes [3, 30, 31].

### Strengths and limitations

The inclusion of family perspectives from diverse socio-economic areas provides valuable insight into the essential components of integrated care for children and young people with ongoing conditions. A considerable strength of this study was the inclusion of families who have traditionally been thought of as experiencing greater barriers to accessing healthcare, including those from the 20% most deprived areas across the UK. However, as the sample was self-selecting, findings may represent the views of those who were more articulate and willing to come forward. In addition, the intervention described here was implemented across one large health setting; it is anticipated that integration at the broader system-level may be more challenging. Furthermore, caregivers interviewed were largely female, with only two fathers participating in interviews.

## Conclusions

Families value specific elements of integrated care, identifying key components that make integrated services high quality. A diverse sample of families with a range of experiences shared important common views about the components needed to integrate health services for children and young people with ongoing conditions. Drawing on our analyses, we make the following recommendations for integrating child health services in ways that are resonant with families’ perspectives:

- Patient empowerment is delivered through personal relationships. Communication technologies can then subsequently supplement the therapeutic relationship.
- Co-ordination between health, social, and education services for children is vital.
- Assessment and understanding of families’ social needs and supporting young people’s autonomy are areas where integrated care may be enhanced.
- Nurses working across organisational boundaries, working as care providers and navigators, may mitigate, but not overcome poorly funded, fragmented services.

## Supplementary Information

**Additional file 1.**

## Data Availability

The datasets generated and/or analysed during the current study are not publicly available in order to protect participants but are available from the corresponding author on reasonable request.

## Abbreviations

CYPHP: Children & Young People’s Health Partnership
IMD: Index of multiple deprivation
