# Supplementary material for: Integrated health Services for Children: a qualitative study of family perspectives
Source: BMC Health Serv Res. 2021 Feb 23;21:167. doi: 10.1186/s12913-021-06141-9 (PMC7901188; doi:10.1186/s12913-021-06141-9)
Supplement: Supplementary file 1 — Additional file 1. [file 12913_2021_6141_MOESM1_ESM.docx]

**Additional File 1.** Interview Guide for Use with Caregivers, Children and Young People

| Introduce researcher and purpose of interview, obtain consent/assent to proceed and to record the conversation  Remind participants that all information remains confidential and they are free to leave at any time | |
| --- | --- |
| **Background Information** | I would like to ask you all a few questions, so we get to know a bit more about each other, and so I can understand which of CYPHP’s new healthcare services you may have experienced.  Could you tell me a little bit about:  Yourself/your child (demographics)  Your/your child’s health (details/knowledge about health condition,  diagnosis, years with diagnosis)  Management of child’s health (caregiver and child’s role; what makes  this manageable/more challenging)  Impact of child’s health  Have you ever discussed these problems with your/your child’s health  provider? (helpful? Is there enough support?) |
| **Experience of Healthcare Prior to CYPHP Evelina London Model of Care** | Can we talk about the healthcare you/your child received before the CYPHP service. When your child was unwell:  Who did you speak to/where did you go for help? (problems accessing  care, what care are they accessing? Support from family/friends)  Did you get all the information you needed (barriers in accessing care)  Good things/problems with care (type of care required; why?) |
| **Accessing the CYPHP Evelina London Model of Care** | Now, let’s talk about how you found out about the CYPHP service you/your child has been receiving:  Finding out about the CYPHP service (referral, online information,  school teacher, text message)  Accessing the service (location, internet, language, what would make  it easier?)  Purpose of the service |
| **Experience of the CYPHP Evelina London Model of Care** | Now, let’s talk about the CYPHP service you/your child has been receiving:  Could you tell me about the CYPHP service that you/your child  experienced (which services, location of services)  Experience of the service from referral to discharge (appointments  attended. If didn’t attend, why not?)  What sort of things did you discuss? (explore self-management  strategies, biopsychosocial care, additional support from  families/friends/health providers, change the way child’s health is  managed?)  Changes in symptom management (change in approach? Techniques used?)  Feelings about discharge from service (confidence in future health  management)  Service feedback (what was useful/not useful, what else would they  like? Different to care previously received?)  Best and worst things about the CYPHP service? (suggestions for  improvement) |
| **Interview Close** | Are there any issues that we have not talked about that you would like to raise? |
